# From Heart to Abdominal Aorta: Integrating Multi-Modal Cardiac Imaging Derived Haemodynamic Biomarkers for Abdominal Aortic Aneurysm Risk Stratification, Surveillance, Pre-Operative Assessment and Therapeutic Decision-Making

**DOI:** 10.3390/diagnostics15192497

**Published:** 2025-10-01

**Authors:** Rafic Ramses, Obiekezie Agu

**Affiliations:** 1 University College London Division of Surgery and Interventional Science, Royal Free Hospital, London NW3 2QG, UK; 2 Department of Vascular Surgery, Royal Free Hospital, London NW3 2QG, UK

**Keywords:** abdominal aortic aneurysm (AAA), wall shear stress (WSS), time-averaged wall shear stress (TAWSS), oscillatory shear index (OSI), relative residence time (RRT), endothelial cell activation potential (ECAP), pulse wave velocity (PWV), blood flow patterns, cardiac MRI (CMR), echocardiography, computational fluid dynamics (CFD)

## Abstract

Recent advances in cardiovascular imaging have revolutionized the assessment and management of abdominal aortic aneurysm (AAA) through the integration of sophisticated haemodynamic biomarkers. This comprehensive review evaluates the clinical utility and mechanistic significance of multiple biomarkers in AAA pathogenesis, progression, and treatment outcomes. Advanced cardiac imaging modalities, including four-dimensional magnetic resonance imaging (4D MRI), computational fluid dynamics (CFD), and specialized echocardiography, enable precise quantification of critical haemodynamic parameters. Wall shear stress (WSS) emerges as a fundamental biomarker, with values below 0.4 Pa indicating pathological conditions and increased risk for aneurysm progression. Time-averaged wall shear stress (TAWSS), typically maintaining values above 1.5 Pa in healthy arterial segments, provides crucial information about sustained haemodynamic forces affecting the vessel wall. The oscillatory shear index (OSI), ranging from 0 (unidirectional flow) to 0.5 (purely oscillatory flow), quantifies directional changes in WSS during cardiac cycles. In AAA, elevated OSI values between 0.3 and 0.4 correlate with disturbed flow patterns and accelerated disease progression. The relative residence time (RRT), combining TAWSS and OSI, identifies regions prone to thrombosis, with values exceeding 2–3 Pa^−1^ indicating increased risk. The endothelial cell activation potential (ECAP), calculated as OSI/TAWSS, serves as an integrated metric for endothelial dysfunction risk, with values above 0.2–0.3 Pa^−1^ suggesting increased inflammatory activity. Additional biomarkers include the volumetric perivascular characterization index (VPCI), which assesses vessel wall inflammation through perivascular tissue analysis, and pulse wave velocity (PWV), measuring arterial stiffness. Central aortic systolic pressure and the aortic augmentation index provide essential information about cardiovascular load and arterial compliance. Novel parameters such as particle residence time, flow stagnation, and recirculation zones offer detailed insights into local haemodynamics and potential complications. Implementation challenges include the need for specialized equipment, standardized protocols, and expertise in data interpretation. However, the potential for improved patient outcomes through more precise risk stratification and personalized treatment planning justifies continued development and validation of these advanced assessment tools.

## 1. Introduction

The landscape of abdominal aortic aneurysm (AAA) research and clinical management is undergoing a profound transformation, driven by the realization that the traditional reliance on maximum aortic diameter is a blunt tool for risk stratification and therapeutic decision-making. The heterogeneity of AAA pathobiology, the unpredictable nature of its progression, and the variable outcomes after repair demand a more refined, mechanistically informed approach. This is where the integration of advanced cardiac imaging modalities, cardiac MRI, echocardiography, and computational fluid dynamics (CFD) models becomes not just relevant, but increasingly essential to future practice.

Cardiac MRI, particularly with quantitative and 4D-flow techniques, has emerged as a powerful modality for interrogating the haemodynamic environment within the aorta. Unlike static anatomical measurements, MRI-derived biomarkers such as wall shear stress (WSS), the oscillatory shear index (OSI), and flow displacement provide a window into the dynamic forces that shape aneurysm biology. These parameters are directly implicated in the mechanotransduction pathways that govern endothelial function, mural inflammation, and extracellular matrix remodelling processes that underlie the initiation and expansion of AAAs [1]. For example, regions of low WSS and high OSI, as revealed by 4D-flow MRI and corroborated by CFD, are consistently associated with increased proteolytic activity, inflammatory infiltration, and wall weakening, all of which are central to aneurysm pathogenesis and growth [2]. The ability of MRI to also capture molecular and wall biomarkers, such as those reflecting inflammation or extracellular matrix degradation, further elevates its role from mere imaging to a tool for in vivo pathophysiological phenotyping.

Echocardiography, especially when augmented with colour Doppler flow (CDF), remains indispensable for its accessibility, real-time assessment, and cost-effectiveness. While often underappreciated in the context of AAA, echo-based modalities can opportunistically detect aneurysms in high-risk populations and provide serial, non-invasive monitoring of aortic morphology and flow dynamics [3,4]. The feasibility of incorporating abdominal aorta imaging into routine echocardiographic protocols is well established, with high rates of successful visualization and detection of clinically significant aneurysms [4]. Moreover, advanced echo techniques, including contrast-enhanced ultrasound, have demonstrated high sensitivity and specificity for detecting endoleaks and other post-repair complications, matching CT angiography while avoiding its attendant risks [5,6]. This positions echocardiography as a frontline tool not only for screening but also for longitudinal surveillance and early detection of adverse remodelling.

CFD models, when integrated with imaging data, offer a unique capability: the simulation and quantification of complex, patient-specific blood flow patterns that are otherwise inaccessible. CFD-derived metrics such as particle residence time, flow stagnation, and recirculation zones have been shown to correlate with mural thrombus burden, wall stress distribution, and regions of accelerated aneurysm growth. These computational biomarkers provide a mechanistic bridge between haemodynamic forces and biological response, enabling the identification of high-risk regions within the aneurysm sac and the prediction of disease trajectory. Importantly, CFD models can be dynamically updated with serial imaging data, allowing for the longitudinal tracking of disease evolution and the impact of interventions; an essential feature given the episodic and non-linear nature of AAA progression [2].

The clinical implications of these advances are profound. Haemodynamic biomarkers derived from multi-modal imaging are not merely surrogate endpoints; they are mechanistically linked to the fundamental processes of AAA pathogenesis, progression, and post-repair outcomes. In the early stages, altered flow patterns characterized by low WSS, high OSI, and increased recirculation initiate a cascade of endothelial dysfunction, inflammatory activation, and matrix degradation [7]. These processes are spatially and temporally heterogeneous, necessitating high-resolution, multi-modal imaging to map disease activity at the tissue and molecular level. Quantitative MRI and positron emission tomography (PET) imaging, for example, can detect molecular signatures of inflammation and proteolysis that are predictive of rapid aneurysm growth and rupture risk, independent of diameter [8]. The integration of circulating biomarkers such as those reflecting immune activation, oxidative stress, and matrix turnover with imaging findings further enhances risk stratification and enables a truly personalized approach to surveillance and intervention (Table 1) [9].

After AAA surgical or endovascular repair, the restoration of physiological haemodynamics is a critical determinant of long-term outcomes. Persistent or recurrent abnormal flow patterns, such as endoleaks or inadequate graft sealing, are major drivers of sac expansion, reintervention, and rupture risk [10]. Advanced imaging biomarkers, such as perivascular fat attenuation (a surrogate for wall inflammation) and dynamic changes in wall motion or flow, have demonstrated strong associations with adverse outcomes and may guide more personalized surveillance and therapeutic strategies. The incorporation of novel metrics like the volumetric perivascular characterization index (VPCI) into clinical practice has shown high accuracy, sensitivity, and specificity for predicting reintervention risk after endovascular aneurysm repair (EVAR), surpassing traditional imaging metrics [10].

This review critically examines haemodynamic biomarkers from cardiac imaging. It assesses the strengths and limitations of various imaging techniques, connecting these biomarkers to the development and progression of AAA. In addition, the review discusses how these biomarkers can be used to predict patient outcomes following surgical or endovascular repair.

**Table 1:** The temporal sequence of haemodynamic and biomarker changes in AAA progression.

Step	Event/Change	Evidence and Reasoning
**1**	Haemodynamic alterations (e.g., decreased WSS, increased OSI, abnormal flow patterns)	In mild-to-moderate aortic dilatation, haemodynamic markers measured by 4D Flow MRI are significantly altered even when circulating biomarkers remain unchanged, suggesting that disturbed flow and shear stress are the earliest detectable changes in AAA pathogenesis [11,12]
**2**	Correlation and subsequent change in circulating biomarkers	As the disease progresses, several circulating biomarkers (e.g., MMPs, collagen markers, D-dimer) begin to correlate with haemodynamic changes, but these biomarker levels do not differ from controls in early disease, indicating that their alteration is a downstream event [13,14]
**3**	Advanced AAA: Both haemodynamic and biomarker changes are present	In more advanced AAA, both haemodynamic and circulating biomarker changes are evident and correlate with disease severity and risk of complications [13,15]

## 2. Wall Shear Stress (WSS) and Derived Metrics

Haemodynamic forces, particularly wall shear stress (WSS) and its derived metrics; time averaged wall shear stress (TAWSS), oscillatory shear index (OSI), endothelial cell activation potential (ECAP), and relative residence time (RRT) play a central role in the pathogenesis, progression, and clinical outcomes of abdominal aortic aneurysm (AAA). These parameters, when measured by advanced cardiac imaging modalities such as 4D flow cardiovascular magnetic resonance (CMR), provide critical insights into the biomechanical environment of the aortic wall and its link to vascular inflammation, aneurysm progression and rupture risk (Table 2).

### 2.1. Definition and Calculation of Wall Shear Stress (WSS)

Wall shear stress (WSS) is defined as the tangential force per unit area exerted by blood flow on the vessel wall, typically measured in Pascals (Pa). It is calculated as the product of blood viscosity and the velocity gradient at the wall, mathematically expressed as WSS = μ(du/dy), where μ is the dynamic viscosity of blood and du/dy is the velocity gradient perpendicular to the wall. WSS is a critical haemodynamic parameter because it influences endothelial cell function, vascular remodelling, and the pathogenesis of vascular diseases, including abdominal aortic aneurysm (AAA).

### 2.2. Physiological Ranges and Pathological Implications of WSS

Low WSS is associated with endothelial dysfunction, inflammation, and matrix degradation, all of which contribute to aneurysm formation, progression, and rupture risk. The physiological range of WSS in large arteries is generally between 1 and 7 Pa, with values below 0.4 Pa considered low and associated with atherogenic risk, while values above 4 Pa may be considered high and potentially damaging to the endothelium [16]. The association between atherogenic risk and damage to the endothelium is sequential and causal; atherogenic risk factors induce endothelial damage, which in turn accelerates atherosclerosis, making endothelial dysfunction both a marker and mediator linking risk to vascular disease [17,18].

### 2.3. Time-Averaged Wall Shear Stress (TAWSS)

Time-averaged wall shear stress (TAWSS) is a scalar metric representing the mean magnitude of WSS over a cardiac cycle. It is calculated as the integral of the absolute value of the instantaneous WSS vector over time, divided by the cycle duration. TAWSS provides a summary of the overall shear environment experienced by the endothelium and is particularly useful in identifying regions exposed to persistently low or high shear. For example, in abdominal aortic aneurysm (AAA) models, regions with TAWSS below 0.4 Pa are considered at higher risk for disease progression and thrombus formation, while healthy arterial segments typically exhibit TAWSS values above 1.5 Pa [16,19].

### 2.4. Oscillatory Shear Index (OSI)

Oscillatory shear index (OSI) quantifies the directional changes in WSS during the cardiac cycle, capturing the extent of flow reversal. OSI is a dimensionless parameter ranging from 0 (unidirectional flow) to 0.5 (purely oscillatory flow with equal forward and reverse components). High OSI values, typically above 0.2, are associated with disturbed flow patterns, endothelial dysfunction, and atherogenesis. In AAA and atherosclerotic regions, OSI values can reach 0.3–0.4, while healthy arteries usually have OSI below 0.1 [20,21].

### 2.5. Relative Residence Time (RRT)

Relative residence time (RRT) is a derived metric that combines TAWSS and OSI to estimate the time blood particles spend near the vessel wall, reflecting the potential for mass transport, particle deposition, and thrombosis. RRT is calculated as (1–2 × OSI)/TAWSS, with units of Pa^−1^ or seconds, depending on normalization. High RRT values indicate prolonged particle residence and are linked to increased risk of atherosclerosis and thrombus formation. In clinical studies, RRT values above 2–3 Pa^−1^ are considered elevated and correlate with disease-prone regions [22,23].

### 2.6. Endothelial Cell Activation Potential (ECAP)

Endothelial cell activation potential (ECAP) is a more recent metric that integrates the effects of low TAWSS and high OSI, providing a measure of the likelihood of endothelial activation and inflammation. ECAP is typically calculated as OSI/TAWSS, with higher values indicating greater risk. ECAP values above 0.2–0.3 Pa^−1^ have been associated with regions of increased endothelial activation, inflammation, and vulnerability to disease [24,25].

### 2.7. Mechanistic Links Between Low WSS and AAA Pathogenesis

The mechanistic link between low WSS and AAA pathogenesis is supported by both in vivo and in vitro studies. Low WSS promotes endothelial cell dysfunction, upregulation of matrix metalloproteinases (MMPs), and inflammatory cell recruitment, leading to extracellular matrix degradation and wall weakening [26]. In vitro models using particle image velocimetry (PIV) have shown that, even at early stages of AAA (≤50% increase in diameter), flow separation and vortex formation result in regions of negative mean WSS and large fluctuations, with the magnitude of WSS dropping to as low as 26% of healthy values [27]. Two distinct regions are observed: a proximal zone of oscillatory, low mean WSS, and a distal zone of large, negative WSS and high gradients, corresponding to sites of flow reattachment and vortex impact. These haemodynamic disturbances are implicated in the spatial heterogeneity of wall degeneration and rupture risk.

### 2.8. Spatial Distribution of WSS and Clinical Correlates in AAA

The spatial distribution of WSS within the aneurysm is also clinically relevant. In a retrospective analysis of 35 AAA patients (13 ruptured, 22 unruptured), the ruptured group had significantly lower WSS at the site of maximal blood flow impact (median 0.549 Pa vs. 1.378 Pa, *p* < 0.001) and at the rupture site itself (median 0.025 Pa, *p* = 0.001) [28]. Maximum diameter and curvature were also associated with rupture risk (OR 1.095, *p* = 0.003 for diameter; OR 1.142E + 10, *p* = 0.012 for curvature), and curvature was negatively correlated with WSS (r = −0.366, *p* = 0.033). These data suggest that both geometric and haemodynamic factors contribute to rupture risk, and that local WSS measurements may identify high-risk regions within the aneurysm [28].

### 2.9. Reproducibility and Imaging Techniques for WSS Measurement

The reproducibility and clinical utility of WSS measurements have been validated in several studies. 4D flow MRI provides robust and reproducible WSS estimates in AAA, with good inter- and intra-observer agreement [29]. Deep learning approaches have also been developed to accelerate WSS prediction from imaging data, achieving a normalized mean absolute error of just 0.362% in WSS prediction across 23 real and 230 synthetic AAA geometries [30]. These advances facilitate the integration of WSS analysis into routine clinical workflows and large-scale studies. While most research has focused on MRI-based techniques, echocardiography remains an important tool for initial AAA assessment and surveillance. However, its role in direct WSS measurement is limited. Doppler ultrasound can estimate blood flow velocities, and with geometric modelling, WSS can be approximated, but this approach is less accurate than MRI or CT-based computational fluid dynamics (CFD) [31]. MRI-based WSS calculations rely on high-resolution velocity mapping and segmentation, while CFD models use patient-specific geometries and boundary conditions derived from imaging to simulate flow and compute WSS distributions [32]. The accuracy of these methods depends on image quality, segmentation precision, and assumptions about blood rheology and flow conditions.

### 2.10. WSS and Outcomes After AAA Repair

The importance of WSS extends to outcomes after AAA repair, including open surgery and endovascular repair (EVAR). Although direct studies linking preoperative WSS to postoperative outcomes are limited, the haemodynamic environment is known to change after repair, potentially influencing graft patency, endoleak risk, and long-term remodelling. In a large registry of 19,964 patients undergoing elective EVAR, those with abnormal preoperative cardiac stress testing (CST) had significantly higher perioperative complications, major adverse cardiac events (MACE), and one-year mortality (OR for MACE 1.59, 95% CI 1.28–1.97; HR for one-year mortality 1.11, 95% CI 1.00–1.23) [33]. While CST is not a direct measure of WSS, it reflects the broader cardiovascular risk profile, which may interact with local haemodynamics to influence outcomes. The integration of WSS analysis with clinical risk factors could enhance perioperative risk stratification and guide surveillance strategies after EVAR.

### 2.11. Dynamic Contrast-Enhanced MRI and Haemodynamic Correlates in AAA

Dynamic contrast-enhanced (DCE) MRI has also been explored as a marker for AAA progression, focusing on wall permeability and neovascularization, which are related to inflammation and matrix degradation. In a study of 27 male AAA patients, DCE-MRI parameters such as the area under the curve (AUC) and the slope of signal increase were significantly associated with historical AAA growth rate, but not with maximum diameter [34]. This suggests that microvascular changes and wall inflammation, which are influenced by local haemodynamics including WSS, play a key role in aneurysm expansion. The spatial heterogeneity of contrast uptake, with higher values at the lateral sides of the AAA, aligns with regions of altered flow and low WSS, supporting the mechanistic link between haemodynamics and wall pathology.

## 3. Pulse Wave Velocity (PWV) as a Marker of Arterial Stiffness in AAA

Pulse wave velocity (PWV) is a haemodynamic parameter reflecting arterial stiffness, and its measurement has become increasingly relevant in the context of AAA risk assessment, progression monitoring, and outcome prediction. The pathophysiological basis for this relationship lies in the chronic inflammation and enzymatic degradation of elastin within the aortic wall, which increases arterial stiffness and, consequently, PWV [35]. This stiffening alters the propagation of the pressure wave generated by cardiac contraction, which can be quantified by PWV using various cardiac imaging modalities, including echocardiography, cardiac MRI, and cardiac CT, though most clinical studies have relied on non-invasive tonometry or plethysmography-based methods [36].

### 3.1. Imaging Modalities and Measurement Techniques for PWV

PWV can be measured regionally or globally. Echocardiography, particularly with pulse wave imaging (PWI), allows for regional assessment of PWV in the abdominal aorta by tracking pulse wave-induced wall motion, providing both quantitative and qualitative data on local arterial stiffness [37]. Cardiac MRI offers high spatial resolution and can measure both global and segmental aortic PWV by phase-contrast velocity mapping, while cardiac CT, though less commonly used for functional assessment, can provide anatomical context and, with advanced techniques, may estimate PWV by tracking contrast bolus transit or wall motion [38]. These imaging-derived parameters are distinct from direct aneurysm imaging (e.g., maximal diameter), focusing instead on the haemodynamic environment that may predispose to AAA development and influence its progression.

### 3.2. Clinical Evidence of Elevated PWV in AAA Patients

Multiple studies have demonstrated that PWV is significantly elevated in patients with AAA compared to healthy controls, with mean differences ranging from 2 to 5 m/s depending on the population and measurement technique. For example, a meta-analysis found that AAA patients had a weighted mean difference in PWV of 2.36 m/s higher than controls, and this elevation was influenced by age, sex, smoking, and hypertension [35]. In a clinical context, aortic PWV measured by the arteriography was 11.5 ± 2.9 m/s in AAA patients versus 7.3 ± 1.6 m/s in healthy individuals, even after adjusting for confounders [39]. These findings are consistent across modalities, with ultrasound-based PWI showing AAA subjects with PWV of 10.54 ± 6.52 m/s compared to 6.03 ± 1.68 m/s in normal [37]. The augmentation index (AIx), another marker of arterial stiffness and wave reflection, is also elevated in AAA, further supporting the link between altered haemodynamics and aneurysm pathology [40].

### 3.3. Prognostic Value of PWV in AAA Risk Stratification and Outcomes

The clinical utility of PWV in AAA extends beyond diagnosis to risk stratification and outcome prediction. Elevated PWV is associated with increased cardiovascular risk and may identify AAA patients at higher risk for adverse events, including aneurysm expansion and rupture [41]. For instance, preoperative brachial-ankle PWV (baPWV) values above 1854 cm/s were associated with a significantly higher rate of sac growth after endovascular aortic repair (EVAR), with a hazard ratio of 2.6 for significant sac enlargement [41]. Similarly, high post-EVAR baPWV (>2100 cm/s) predicted poor aneurysm shrinkage and increased cardiovascular events, with five-year event-free rates dropping from 73% in those with lower PWV to 46% in those with higher PWV [42].

### 3.4. Pathophysiological Basis Linking PWV and AAA

The link between increased PWV and AAA development is rooted in the loss of aortic wall elasticity due to matrix degradation, inflammation, and smooth muscle cell apoptosis. This results in a stiffer aorta, which not only predisposes to aneurysm formation by increasing wall stress but also accelerates aneurysm growth by perpetuating adverse haemodynamic forces [38]. For example, in a hypothetical patient with a 45 mm AAA and a PWV of 13 m/s measured by cardiac MRI, the elevated stiffness would indicate both a higher risk of concurrent coronary artery disease and a greater likelihood of rapid aneurysm expansion, necessitating closer surveillance and potentially earlier intervention [39]. Conversely, a patient with a similar aneurysm size but a PWV of 8 m/s might be considered at lower risk for progression, assuming other risk factors are controlled.

### 3.5. Impact of AAA Repair on PWV and Postoperative Outcomes

The relationship between PWV and AAA outcomes after surgery or EVAR is particularly important. EVAR itself can acutely increase aortic stiffness, as evidenced by a rise in post-procedural PWV, which may reflect the loss of native aortic compliance due to stent graft implantation [42]. Patients with persistently high PWV after EVAR are more likely to experience poor aneurysm shrinkage and higher rates of cardiovascular events, suggesting that PWV monitoring could inform postoperative management and secondary prevention strategies. For example, a patient with a pre-EVAR baPWV of 2000 cm/s who remains above 2100 cm/s post-EVAR would be at increased risk for sac growth and adverse events, warranting more aggressive risk factor modification and imaging follow-up [42].

## 4. Blood Flow Patterns

Blood flow within AAAs is characterized by complex, three-dimensional patterns that are fundamentally distinct from the flow observed in healthy aortas.

### 4.1. Classification of Flow Patterns

The classification of these patterns primarily into laminar, vortical, and helical flows has become increasingly relevant for understanding the biomechanical environment of AAAs and its direct association with rupture risk.

### 4.2. Vortical Flow: Origins and Subtypes

Vortical flow, characterized by the presence of one or more recirculating eddies, is a hallmark of disturbed haemodynamics in AAAs. Vortices arise due to abrupt changes in vessel geometry, such as the transition from the normal aorta to the aneurysmal sac, and are further influenced by asymmetry, wall irregularities, and the presence of ILT. CFD studies have classified vortical flow patterns in AAAs into subtypes based on the number and organization of vortices. For instance, a recent case–control study introduced a three-type classification: Type I (non-helical main flow with multiple vortices), Type II (non-helical main flow with a single vortex), and Type III (helical main flow with helical vortices) [43]. 

### 4.3. Association of Flow Patterns with Rupture Risk

The prevalence of these patterns is not uniform across patient populations; in a cohort of 106 patients, Type III (helical) flow was observed in 60.4% of ruptured AAAs compared to only 15.1% of intact AAAs (*p* < 0.001), indicating a strong association between helical-vortical flow and rupture risk [43].

### 4.4. Helical Flow: Characteristics and Impact on Wall Stress

Helical flow, defined by the presence of a spiral or corkscrew-like motion of blood, represents a highly disturbed regime that is particularly relevant in the context of large, asymmetric, or tortuous AAAs. Helical flow is not merely a geometric curiosity; it is associated with significant alterations in local haemodynamic forces. The same case–control study found that Type III (helical) flow conferred an odds ratio (OR) of 10.22 (95% CI: 3.43–30.49) for rupture compared to non-helical patterns [43]. This dramatic increase in risk is thought to arise from the unique wall stress distributions generated by helical flow, including regions of both abnormally high and low WSS, as well as elevated oscillatory shear index (OSI).

### 4.5. Intraluminal Thrombus (ILT): Modulation of Flow and Rupture Risk

The role of ILT in modulating flow patterns and rupture risk is complex and context dependent. While a larger ILT burden is associated with more laminar flow and reduced wall stress in some models [44,45], rapid ILT growth and increased ILT volume over time correlate with higher peak wall rupture index (PWRI) and AAA volume growth, suggesting a dynamic interplay between thrombus formation, flow disturbance, and wall weakening [46]. Furthermore, the material properties of both the arterial wall and ILT influence the transmission of haemodynamic forces; for example, increasing ILT burden can lower effective wall stresses, but this protective effect may be offset by the promotion of hypoxia and proteolytic activity within the wall [45].

## 5. Cardiovascular Imaging Modalities for Haemodynamic Biomarkers

The integration of advanced cardiovascular imaging modalities specifically cardiac MRI flow, echocardiography, and computational fluid dynamics (CFD) has enabled precise quantification of haemodynamic biomarkers such as wall shear stress (WSS), its derivatives, and complex flow patterns, which are vital in elucidating the mechanisms underlying AAA development and progression.

### 5.1. Cardiac MRI

CMR (cardiac magnetic resonance imaging), through time-resolved phase-contrast imaging, allows for the direct quantification of blood flow velocities and the derivation of WSS vectors along the aortic wall. In the context of AAA, regions of abnormally low WSS (<0.4 N/m^2^) and elevated OSI have been consistently mapped to sites of aneurysmal initiation and expansion. These haemodynamic environments promote endothelial dysfunction, upregulation of matrix metalloproteinases (MMPs), and inflammatory cell infiltration, all of which are mechanistically implicated in medial degeneration and extracellular matrix breakdown. Recent clinical studies leveraging CMR-based flow mapping have demonstrated that patients with progressive AAA exhibit a 25–40% reduction in mean WSS within the aneurysmal sac compared to non-aneurysmal segments, with corresponding increases in OSI and flow recirculation zones. These findings are corroborated by proteomic analyses showing elevated circulating levels of MMP-9, elastin degradation products, and inflammatory mediators in patients with low WSS regions, supporting a direct mechanistic link between CMR-derived haemodynamics and molecular drivers of AAA development [47,48].

#### 5.1.1. Haemodynamic Biomarkers and AAA Progression

Beyond the initial formation, the progression of AAA is governed by dynamic changes in local flow patterns, which can be serially monitored using CMR. Longitudinal studies have shown that a decrease in WSS of more than 15% per year, as measured by CMR, is associated with a twofold increase in aneurysm growth rate and a 1.8-fold higher risk of rupture or rapid expansion. These haemodynamic biomarkers outperform traditional risk factors such as baseline diameter or smoking status in multivariate models, underscoring their prognostic value. The VASCUL-AID-RETRO study, a large-scale European initiative, is currently integrating CMR-derived haemodynamic parameters with clinical and multi-omics data to develop AI-driven predictive models for AAA progression. Early results indicate that the inclusion of CMR-based WSS and flow complexity indices improves the accuracy of progression risk stratification by 18–22% over models relying solely on clinical and biochemical markers [47]. This integration is particularly valuable in patients with small or borderline aneurysms, where conventional diameter thresholds fail to capture the true risk of adverse events.

#### 5.1.2. Endothelial Dysfunction and Cellular Mechanisms

Low and oscillatory WSS, as quantified by CMR, induce a pro-inflammatory endothelial phenotype characterized by increased expression of adhesion molecules (VCAM-1, ICAM-1), enhanced leukocyte recruitment, and local production of reactive oxygen species. These processes facilitate the transmural infiltration of macrophages and lymphocytes, which secrete proteases and cytokines that degrade the structural integrity of the aortic wall. Furthermore, CMR-based mapping of flow vortices and helicity has revealed that complex, multidirectional flow within the aneurysmal sac correlates with spatial heterogeneity in wall stress and focal areas of mural thrombus formation. These biomechanical insights, derived from high-resolution CMR, provide a direct link between flow-derived biomarkers and the cellular and molecular events driving AAA progression.

#### 5.1.3. CMR in Post-Intervention Surveillance and Outcomes

CMR flow quantification offers unique advantages in the assessment of both open surgical and EVAR outcomes. Post-intervention, the restoration of physiologic WSS and normalization of flow patterns are critical determinants of long-term success. CMR enables non-invasive, radiation-free surveillance of these parameters, allowing for the early detection of endoleaks, graft migration, or persistent flow disturbances that may predispose to sac enlargement or rupture. Clinical studies have demonstrated that patients with persistent low WSS or abnormal flow complexity after EVAR have a 27% higher rate of sac expansion and a 19% increased risk of secondary interventions compared to those with normalized haemodynamics. Moreover, CMR-derived biomarkers can identify subclinical graft-related complications before they manifest as changes in sac diameter, facilitating timely intervention and improved outcomes.

#### 5.1.4. Integration of CMR Haemodynamics with Circulating Biomarkers

The integration of CMR haemodynamic data with circulating biomarkers further refines risk stratification and mechanistic understanding. For example, elevated levels of galectin-3, pentraxin-3, and interleukin-6 biomarkers associated with inflammation and extracellular matrix remodelling have been shown to correlate with regions of low WSS and high OSI on CMR, independent of aneurysm size. In a recent cohort, the addition of these biomarkers to CMR-derived haemodynamic indices improved the prediction of rapid AAA growth and rupture risk by 15–20%, particularly in patients with small or moderate-sized aneurysms [49].

#### 5.1.5. Impact of Haemodynamic Normalization on Molecular Pathways

Mechanistically, the restoration of laminar flow and normalization of WSS following successful surgical or endovascular repair are associated with downregulation of pro-inflammatory and proteolytic pathways. CMR studies have shown that patients with complete exclusion of the aneurysmal sac and re-establishment of physiologic flow exhibit a 30–35% reduction in circulating MMP-9 and elastin degradation products within six months post-procedure. Conversely, persistent flow disturbances detected by CMR are linked to ongoing biomarker elevation and adverse remodelling, highlighting the importance of haemodynamic normalization as a therapeutic endpoint [50].

#### 5.1.6. Advanced CMR Applications in Cardiac Remodelling and Risk Prediction

The application of advanced CMR techniques extends beyond the aorta to include cardiac remodelling and ventricular-vascular interactions in AAA patients. It allows for the assessment of left ventricular (LV) and right ventricular (RV) function, myocardial fibrosis, and strain patterns, which are increasingly recognized as contributors to perioperative risk and long-term outcomes. For instance, CMR-based quantification of LV mass and fibrosis has been shown to predict adverse cardiac events in AAA patients undergoing repair, with a 22% increase in event rates among those with elevated myocardial extracellular volume fraction [51,52].

### 5.2. Echocardiography

The integration of echocardiographic flow pattern analysis into the study of AAA pathophysiology and management represents a sophisticated frontier in cardiovascular imaging. While echocardiography is traditionally focused on cardiac chambers, its advanced modalities especially Doppler and echo particle image velocimetry (Echo PIV) enable the quantification of complex haemodynamic phenomena that are mechanistically linked to AAA biology and clinical outcomes (Table 3).

#### 5.2.1. Haemodynamic Forces and Vascular Remodelling in AAA

The development of AAA is fundamentally a process of maladaptive vascular remodelling, driven by chronic alterations in haemodynamic forces. Pulsatile flow from the heart, as characterized by echocardiography, imparts cyclical wall shear stress (WSS) and pressure on the aortic wall. In the healthy aorta, laminar flow and physiologic WSS maintain endothelial homeostasis. However, as the aorta dilates and the geometry becomes irregular, as in early AAA, flow patterns become increasingly disturbed in the aneurysmal sac [53].

#### 5.2.2. Evolution of Intra-Aneurysmal Flow Patterns

As AAA progresses, the complexity of intra-aneurysmal flow increases. Time-resolved flow mapping reveals that during systolic acceleration, a central jet traverses the aneurysm, while during deceleration and diastole, large-scale vortices and helical flow patterns dominate the sac [54]. These recirculating flows create zones of low velocity and prolonged residence time, which are associated with thrombus formation and further wall weakening.

#### 5.2.3. Wall Shear Stress Heterogeneity and Aneurysm Geometry

The spatial heterogeneity of WSS, as detected by high-resolution echocardiographic techniques, correlates with regions of accelerated wall remodelling and inflammation. Notably, the asymmetry of the aneurysm and the size of the neck critically modulate these flow patterns, with more asymmetric and wide-necked aneurysms exhibiting greater flow disturbance and lower WSS [55].

#### 5.2.4. Advanced Echocardiographic Modalities: Echo PIV and Doppler Techniques

The translation of these haemodynamic insights into clinical practice is being accelerated by the development of Echo PIV systems, which allow real-time, non-invasive measurement of multi-dimensional velocity and shear stress components in the aorta and heart [53]. Echo PIV can capture vortex rings, shear layers, and stagnation zones within in vitro AAA models, with high concordance to Doppler measurements. This enables the quantification of flow complexity and WSS heterogeneity, providing a direct link between cardiac output patterns and the local haemodynamic environment of the AAA. Such detailed flow mapping is clinically unfeasible with conventional Doppler alone, highlighting the transformative potential of advanced echocardiographic modalities.

In vitro flow visualization and laser Doppler anemometry studies demonstrate that stent-grafts introduce new regions of flow separation, low velocity, and recirculation, particularly at the graft ends and within the iliac limbs [56]. These altered flow patterns are associated with thrombosis and intimal hyperplasia, which can compromise graft patency and long-term outcomes. The stent struts themselves can cause localized flow disturbances, but the overall three-dimensional flow structure is determined by the interplay between the device and the native aortic geometry. Importantly, improvements in stent-graft design that minimize unfavourable flow patterns have the potential to reduce these complications.

### 5.3. Computational Fluid Dynamics (CFD)

Computational fluid dynamics (CFD) has become an indispensable tool for extracting haemodynamic biomarkers that are directly relevant to the pathogenesis, progression, and treatment of AAA. Unlike traditional imaging, CFD enables the quantification of complex, patient-specific flow phenomena and mass transport processes that are otherwise inaccessible, providing a mechanistic window into the disease at a molecular and cellular level (Table 4).

#### 5.3.1. Patient-Specific Modelling and Simulation Techniques

CFD models, constructed from high-resolution imaging such as 4D-flow MRI or CT angiography, allow for the simulation of blood flow, WSS, OSI, and the transport of O_2_ and NO within the aortic lumen and wall. These simulations reveal that regions of low time-averaged WSS and high OSI are consistently found in aneurysmal segments, distinguishing them from healthy aorta.

#### 5.3.2. Haemodynamic Biomarkers and Disease Mechanisms

Such haemodynamic environments are not merely descriptive; they are mechanistically linked to endothelial dysfunction, altered NO production, and hypoxia, all of which are central to AAA pathogenesis. For example, CFD-based mass transfer models have shown that NO production, which is driven by local WSS, is spatially heterogeneous in aneurysmal tissue, with clear distinctions from healthy controls. This spatial variation in NO and O_2_ distribution, and the identification of hypoxic regions due to mass transfer limitations, provide direct biochemical markers that reflect the risk of disease progression and rupture [57].

#### 5.3.3. CFD in Tracking AAA Progression

In terms of progression, CFD enables the longitudinal tracking of haemodynamic biomarkers in patient-specific geometries. By simulating the evolving flow field as the aneurysm enlarges, CFD can quantify changes in WSS, OSI, and endothelial cell activation potential (ECAP). These parameters are not static; as the aneurysm grows, the flow becomes more disturbed, WSS decreases further, and OSI and ECAP increase, creating a feedback loop that perpetuates wall degeneration.

#### 5.3.4. Predictive Modelling of AAA Growth and Rupture Risk

Importantly, CFD-based predictive models have demonstrated the ability to differentiate between fast- and slow-growing AAAs with high accuracy (Table 5), using these haemodynamic biomarkers as input features [58,59].

#### 5.3.5. CFD Applications in AAA Treatment and Device Optimization

CFD also plays a vital role in the assessment and optimization of AAA treatment, particularly endovascular repair. By simulating post-intervention flow, CFD can evaluate the impact of stent design and placement on local haemodynamics, including the restoration of physiological WSS and the minimization of flow disturbances that could lead to endoleak or continued aneurysm expansion. Advanced CFD-based optimization algorithms iteratively adjust device parameters, such as stent porosity, to achieve haemodynamic conditions that are known to reduce rupture risk and improve long-term outcomes. These simulations can be tailored to patient-specific geometries, enabling truly personalized device design and procedural planning [60]. Furthermore, CFD-derived biomarkers serve as early indicators of suboptimal haemodynamic environments post-treatment, guiding surveillance and potential reintervention before clinical complications arise.

#### 5.3.6. The Role of the Windkessel Effect in CFD Simulations

The Windkessel effect, representing the compliance and resistance of the peripheral vasculature, is fundamental for accurately modelling the pulsatile nature of blood flow in the aorta and its branches. In CFD simulations of AAA, the Windkessel model is commonly used as an outlet boundary condition to replicate the physiological pressure and flow waveforms observed in vivo. The three-element Windkessel model (WK3) incorporates characteristic impedance, compliance, and peripheral resistance, allowing CFD to capture the dynamic interplay between the heart’s pulsatile output and the vascular system’s buffering capacity. This is particularly important in AAA, where altered aortic compliance and downstream resistance can significantly affect local flow patterns, WSS, OSI, and pressure gradients, biomarkers intimately linked to disease mechanisms (Table 6). By calibrating Windkessel parameters using patient-specific data (e.g., from 4D Flow-MRI or ultrasound), CFD simulations can reproduce realistic pressure and flow distributions, enabling the identification of regions prone to low WSS and high OSI, which are associated with endothelial dysfunction, inflammation, and matrix degradation in AAA pathogenesis [61,62].

#### 5.3.7. Windkessel Effect and AAA Progression

During AAA progression, the Windkessel effect modulates the propagation of pressure and flow waves, influencing the spatial and temporal distribution of haemodynamic forces. CFD models that incorporate Windkessel boundary conditions can simulate how changes in aortic compliance (due to aneurysmal dilation or wall degeneration) alter the transmission and reflection of pulsatile energy. This, in turn, affects biomarkers such as peak wall stress (PWS) and relative residence time (RRT), which are predictive of aneurysm growth, thrombus formation, and rupture risk [63].

#### 5.3.8. Windkessel Effect in Post-Treatment Assessment

In the context of treatment, especially endovascular repair, the Windkessel effect remains critical. Post-intervention, the compliance of the aorta is often reduced due to stent graft placement, altering the Windkessel properties and thus the downstream pressure and flow environment. CFD simulations that account for these changes can predict the impact on local haemodynamics, helping to identify risks such as endoleak, graft migration, or continued aneurysm expansion. Optimizing device design and placement using CFD with Windkessel boundary conditions ensures that physiological flow and pressure waveforms are restored as closely as possible, minimizing adverse outcomes [61].

### 5.4. Imaging Biomarkers of Wall Inflammation

Imaging biomarkers of wall inflammation, such as the volumetric perivascular characterization index (VPCI), are increasingly recognized for their ability to noninvasively assess vascular and cardiac wall inflammation using advanced cardiac imaging modalities. These biomarkers are not generic markers of disease but are derived from specific imaging features that reflect underlying inflammatory processes in the vessel or cardiac wall, often before overt structural changes or clinical events occur.

VPCI is a quantitative imaging biomarker obtained from CTA. It works by mapping spatial changes in perivascular fat attenuation, which is sensitive to the inflammatory state of the adjacent vessel wall. Inflammation in the vessel wall alters the composition and density of the surrounding perivascular adipose tissue, which can be detected as changes in Hounsfield units (HU) on CT images. By analyzing these changes over time, VPCI provides a dynamic, non-invasive measure of wall inflammation. In the context of AAA and post-endovascular aortic repair (EVAR), upward trends in VPCI trajectories have been strongly associated with increased risk of reintervention, while downward trends are seen in stable or regressing cases [10]. This makes VPCI a potentially powerful tool for monitoring patients after EVAR and for risk stratification before intervention (Table 7).

The technical process for obtaining VPCI from cardiac imaging involves high-resolution, contrast-enhanced CT scans. The perivascular adipose tissue is segmented, and its attenuation is measured in predefined regions around the vessel of interest. Advanced image analysis software is used to calculate the VPCI by tracking changes in fat attenuation over serial scans. This approach is not limited to the aorta; similar principles are applied in coronary imaging, where pericoronary adipose tissue (PCAT) attenuation measured by coronary CTA serves as a biomarker for coronary inflammation. Studies have shown that increased PCAT attenuation is associated with higher risk of coronary artery disease, plaque instability, and adverse cardiac events [64,65]. The pericoronary fat attenuation index (FAI), for example, is a validated metric derived from routine coronary CTA that quantifies local inflammation and predicts future cardiac mortality independently of plaque burden or calcium score [66].

## 6. Cardiac Haemodynamic Biomarkers in Pre-Operative Risk Assessment

In the preoperative setting for AAA repair, WSS and its derivatives have emerged as powerful, quantifiable biomarkers for risk stratification and outcome prediction beyond traditional metrics like aneurysm diameter. Recent prospective and computational studies have demonstrated that low baseline WSS, particularly values below 0.4 Pa, is independently associated with both accelerated AAA expansion and a higher incidence of aneurysm-related events, including rupture and the need for surgical repair. In a cohort of nearly 300 patients, those with low WSS experienced a 44% rate of aneurysm-related events, compared to 27–29% in higher WSS groups, and this association persisted after adjustment for established risk factors [67]. Furthermore, low WSS was linked to a measurable increase in annual aneurysm growth rate, highlighting its value as a dynamic predictor of disease progression and the need for timely intervention (Table 8).

### 6.1. Prognostic Value of Wall Shear Stress (WSS) in AAA

The predictive power of WSS is further enhanced when combined with volumetric imaging data. Models that integrate both baseline WSS and lumen volume outperform maximal diameter alone in forecasting aneurysm enlargement, especially in AAAs smaller than 50 mm. In these cases, the area under the receiver operating characteristic curve (AUC) for combined models reached 0.79, compared to just 0.53 for diameter-based assessment, indicating a substantial improvement in discriminative ability for identifying patients at risk of rapid progression [32]. This is particularly relevant for preoperative planning, as it allows for more precise identification of patients who may benefit from earlier or more aggressive intervention.

### 6.2. Integration of WSS with Volumetric Imaging for Risk Prediction

Beyond mean WSS, derivatives such as TAWSS, OSI, and RRT provide additional granularity. High OSI and RRT, which reflect disturbed and stagnant flow, are associated with increased inflammatory activity and wall degeneration, both of which are implicated in AAA growth and rupture. Advanced imaging modalities, such as 4D flow CMR, enable comprehensive mapping of these parameters. In AAA patients, markedly lower peak WSS and higher RRT are observed not only in the aneurysmal segment but throughout the aorta, highlighting the systemic nature of haemodynamic derangement in this population [19]. These findings suggest that preoperative assessment of WSS, OSI, and RRT can identify patients with globally abnormal haemodynamics who may be at higher risk for adverse outcomes, both aneurysm-related and cardiovascular.

### 6.3. Role of WSS Derivatives: TAWSS, OSI, and RRT

Patient-specific computational fluid dynamics (CFD) and fluid–structure interaction (FSI) models, informed by high-resolution imaging (CT, CMR, or 3D ultrasound), allow for individualized risk assessment. These models reveal that anatomical features such as aortic tortuosity and the presence of intraluminal thrombus can further modulate local WSS and its derivatives, influencing rupture risk and postoperative outcomes. For example, high aortic torsion is associated with lower TAWSS, higher OSI, and more disordered flow, all of which increase rupture risk. After EVAR, WSS and flow velocities typically increase, but persistent hypertension can elevate OSI and TAWSS, emphasizing the need for meticulous blood pressure control in the perioperative period [68,69].

## 7. Integrating Cardiac Haemodynamic Biomarkers into AAA Imaging Protocols

The integration of cardiac haemodynamic biomarkers, particularly WSS and its derivatives, into the clinical management of AAA represents a frontier in vascular medicine.

### 7.1. Current AAA Imaging Protocols and Clinical Guidelines

Current AAA imaging protocols in Europe and the US are primarily based on anatomical criteria most notably, maximum aortic diameter using ultrasound for screening and computed tomography angiography (CTA) or magnetic resonance angiography (MRA) for preoperative planning, postoperative surveillance, and post-EVAR follow-up. These guidelines, such as those from the European Society for Vascular Surgery (ESVS) and the Society for Vascular Surgery (SVS), recommend one-time screening for men aged 65 or older, with surveillance intervals determined by aneurysm size and growth rate. Intervention is generally considered when the diameter exceeds 5.5 cm in men or 5.0 cm in women, or if rapid expansion or symptoms occur. Post-EVAR, imaging is performed at 1 month, 6 months, 12 months, and annually thereafter, focusing on endoleak detection and sac size changes.

### 7.2. Limitations of Diameter-Based Risk Stratification

Despite the utility of these protocols, reliance on diameter alone is increasingly recognized as insufficient for individualized risk stratification. Many AAAs rupture below the intervention threshold, while others remain stable despite exceeding it. This has driven interest in haemodynamic biomarkers (WSS, TAWSS, OSI, RRT, and ECAP) which reflect the biomechanical environment influencing aneurysm growth, thrombus formation, and rupture risk [26,70]. These parameters, derived from advanced imaging modalities such as 4D flow MRI and computational fluid dynamics (CFD) based on patient-specific imaging, provide spatially resolved, physiologically relevant data that can augment anatomical assessment.

### 7.3. WSS Timing and Patient Selection in AAA Management

WSS measurement is most relevant in patients with small to medium-sized AAAs who are under surveillance for disease progression. In these patients, WSS and related parameters such as TAWSS, OSI, and RRT have been shown to correlate with aneurysm growth and the risk of rupture. Specifically, lower WSS and higher RRT are associated with increased AAA expansion and may serve as early markers for disease progression, especially in aneurysms less than 50 mm in diameter. In a prospective registry, baseline WSS combined with lumen volume outperformed maximal diameter alone in predicting aneurysm enlargement at one year [19,71].

The timing of WSS measurement is crucial. Preoperative assessment allows for risk stratification and may influence the decision to intervene, especially in cases where the aneurysm does not meet traditional size criteria but exhibits haemodynamic features suggestive of higher risk. Serial WSS measurements during follow-up can track changes in haemodynamic stress, providing dynamic information about aneurysm behaviour. Longitudinal studies have demonstrated that both maximum WSS and peak wall rupture index independently predict AAA volume growth, supporting the utility of repeated WSS assessment over time [19,46].

After endovascular or open repair, the role of WSS measurement is less well established but potentially valuable. Post-repair, altered flow dynamics can predispose individuals to complications such as endoleak or graft failure. While direct evidence linking WSS measurement to endoleak prediction is limited, regions of low WSS and high wall pressure have been shown to correspond to sites of potential rupture and may similarly indicate areas at risk for endoleak formation. CFD studies suggest that surveillance of areas with the lowest WSS post-repair could help identify patients at risk for adverse outcomes, although more research is needed to validate this approach in the postoperative setting [25,72].

Certain patient subgroups may particularly benefit from WSS assessment. These include individuals with complex aneurysm morphology, significant intraluminal thrombus (ILT), or rapid aneurysm growth despite small diameter. Low WSS regions are associated with ILT deposition, which in turn promotes further aneurysm expansion and wall weakening. Patients with substantial ILT or evidence of rapid ILT accumulation may therefore warrant more detailed haemodynamic evaluation, as WSS measurement can help clarify the interplay between thrombus formation and aneurysm progression [73,74].

### 7.4. Challenges in Clinical Integration of Haemodynamic Biomarkers

The translation of these biomarkers into clinical protocols faces several challenges. First, the acquisition and processing of haemodynamic data require high-resolution imaging (e.g., 4D flow MRI or high-quality CTA for CFD modelling), specialized software, and expertise in image segmentation and flow simulation [1,70]. Standardization of acquisition protocols, post-processing pipelines, and parameter thresholds is lacking, which limits reproducibility and comparability across centres [1,12]. Secondly, while cross-sectional and retrospective studies support the association between haemodynamic parameters and AAA progression or rupture, prospective longitudinal studies validating their predictive value and clinical utility are sparse [25]. Thirdly, the incremental benefit of these biomarkers over established risk factors (diameter, growth rate, symptoms) in guiding intervention or surveillance intervals remains to be demonstrated in large, multicentre cohorts [1].

### 7.5. Augmenting Existing Protocols: Potential Clinical Applications

In terms of integration with current guidelines, haemodynamic biomarkers are best positioned to augment, rather than replace, existing imaging modalities and protocols in the near term. For screening, the population-based approach using ultrasound remains appropriate due to its accessibility, cost-effectiveness, and established mortality benefit. However, in patients with detected AAAs, especially those with borderline diameters or atypical growth patterns, haemodynamic assessment could refine risk stratification. For example, patients with small but haemodynamically high-risk AAAs (low TAWSS, high OSI/RRT) might warrant closer surveillance or earlier intervention, while those with large but biomechanically stable AAAs could be monitored more conservatively [70,73]. In the preoperative setting, haemodynamic mapping can inform procedural planning by identifying regions at risk for rupture or thrombus-related complications [25,26]. Postoperatively, especially after EVAR, haemodynamic biomarkers could help detect regions prone to endoleak, sac expansion, or ILT formation, potentially guiding adjunctive therapy or reintervention.

### 7.6. Considerations for Implementation

The optimal timing for haemodynamic assessment depends on the clinical context. At diagnosis, baseline haemodynamic mapping can establish a risk profile. During surveillance, repeat assessment at intervals aligned with anatomical imaging (e.g., annually or at 6–12 month intervals for AAAs > 4.0 cm) can track changes in biomechanical risk, particularly if there is evidence of rapid growth or morphological change [1,12]. After EVAR, haemodynamic imaging at 1 month and 12 months, in parallel with standard CTA or MRA, could identify early biomechanical predictors of adverse outcomes [73,75]. Importantly, the integration of haemodynamic biomarkers should be guided by evidence of clinical benefit, cost-effectiveness, and feasibility within existing workflows.

## 8. Conclusions

The integration of multi-modal cardiovascular imaging, including cardiac MRI, echocardiography, and computational fluid dynamics, is revolutionizing the assessment and management of abdominal aortic aneurysms. The quantification of haemodynamic biomarkers, such as wall shear stress, oscillatory shear index, and pulse wave velocity, provides crucial insights into the biomechanical environment driving disease progression and treatment outcomes. The continued development and validation of these advanced imaging tools hold the promise of improved patient risk stratification, personalized surveillance, and optimized therapeutic decision-making for individuals with abdominal aortic aneurysms. Future prospective multicenter randomized clinical studies are essential to validate the predictive value of haemodynamic biomarkers from cardiac MRI and echocardiography for AAA progression, rupture risk, and post-repair outcomes. Such studies would enable the establishment of causal relationships and reproducibility of these biomarkers, ultimately supporting their integration into multifactorial risk models for personalized AAA management.

## Figures and Tables

**Table 2:** Summary of wall shear stress–derived parameters, mechanistic insights, and clinical applications in abdominal aortic aneurysm.

Category Metric	Definition Formula	Physiological Clinical Range	Mechanistic Role in AAA	Clinical Findings	Measurement Methods	Measurement Advances	Clinical Application
**Wall Shear Stress (WSS)**	Tangential force per unit area exerted by blood flow on vessel wall; WSS = μ(du/dy)	1–7 Pa (normal); <0.4 Pa (low, high risk); >4 Pa (high, potentially damaging)	Influences endothelial function, vascular remodelling, AAA pathogenesis; low WSS promotes dysfunction, inflammation, matrix degradation	Low WSS linked to AAA progression, rupture risk, and wall weakening; lower WSS at rupture sites	4D flow MRI, CFD, Doppler ultrasound (less accurate)	4D flow MRI and deep learning models for accurate WSS prediction, reducing computational time compared to CFD	WSS and geometry (diameter, curvature) together improve rupture risk prediction; low WSS predicts AAA expansion and events;
**Time-Averaged WSS (TAWSS)**	Mean WSS over cardiac cycle	<0.4 Pa (high risk); >1.5 Pa (healthy)	Identifies regions with persistently low/high shear, risk for thrombus formation	Low TAWSS associated with AAA progression, higher rupture risk	4D flow MRI, CFD	Deep learning models accelerate TAWSS calculation; validated against CFD	TAWSS included in multivariable models for rupture prediction; low TAWSS is an independent risk factor for AAA events
**Oscillatory Shear Index (OSI)**	Quantifies directional changes in WSS; 0 (unidirectional) to 0.5 (oscillatory)	>0.2 (disturbed flow, high risk); <0.1 (healthy)	High OSI linked to disturbed flow, endothelial dysfunction, atherogenesis	High OSI increases rupture risk, especially in 50–65 mm AAAs	4D flow MRI, CFD	OSI can be computed from imaging data using advanced algorithms	OSI is a risk factor for rupture in medium-sized AAAs; included in predictive models
**Relative** **Residence Time (RRT)**	(1–2 × OSI)/TAWSS	>2–3 Pa^−1^ (elevated, high risk)	High RRT indicates prolonged particle residence, risk of atherosclerosis and thrombus	High RRT correlates with disease-prone regions, abnormal AAA haemodynamics	4D flow MRI, CFD	RRT derived from TAWSS and OSI; can be mapped across aorta for risk stratification	RRT is a marker for abnormal AAA haemodynamics and may improve risk stratification
**Endothelial Cell Activation** **Potential (ECAP)**	OSI/TAWSS	>0.2–0.3 Pa^−1^ (high risk)	Integrates low TAWSS and high OSI, predicts endothelial activation/inflammation	High ECAP linked to increased inflammation, vulnerability	4D flow MRI, CFD	Calculated from standard WSS; practical for research and clinical studies	ECAP identifies regions of increased endothelial activation and inflammation
**Geometric Factors**	Maximum diameter, curvature, aspect ratio	Larger diameter, higher curvature, deeper sac, smaller neck width = lower WSS, higher risk	Geometry influences local WSS, rupture risk	Curvature negatively correlates with WSS; geometric analysis improves risk prediction	CT angiography, CFD	Automated 3D reconstructions and CFD enable detailed geometric and haemodynamic analysis	Combined geometric and WSS analysis outperforms diameter alone for rupture prediction

**Table 3:** Mechanistic links between AAA flow patterns and echocardiographic assessment.

Mechanistic Feature	Role in AAA	Echocardiographic/Imaging Link
**Vortex formation and recirculation**	Promotes thrombus, wall weakening	Echo PIV, Doppler, CFD validation
**Low/oscillatory wall shear stress**	Drives inflammation, matrix degradation	Doppler-derived WSS, Echo PIV
**Flow stagnation zones**	Predicts rapid AAA growth	CFD, Echo PIV, radiomics
**Post-EVAR altered flow**	Linked to thrombosis, intimal hyperplasia	Flow visualization, Doppler
**Windkessel effect, pulse wave reflections**	Modulates intra-aneurysmal stress	Echo-derived cardiac output, advanced modelling

**Table 4:** CFD biomarkers and their mechanistic and clinical relevance in AAA.

Biomarker/Parameter	Mechanistic Link to AAA	Application in Pathogenesis, Progression, and Treatment
**Wall Shear Stress (WSS)**	Low WSS promotes endothelial dysfunction, inflammation, and matrix degradation	Identifies regions at risk for disease initiation and growth
**Oscillatory Shear Index (OSI)**	High OSI indicates disturbed, reversing flow, linked to wall degeneration	Correlates with rapid expansion and rupture risk
**Endothelial Cell Activation Potential (ECAP)**	Reflects endothelial response to non-physiological shear	Predicts areas of high risk for progression
**Nitric Oxide (NO) Distribution**	Altered NO due to WSS changes affects vascular tone and remodelling	Biomarker for disease activity and rupture risk
**Oxygen (O_2_) Distribution/Hypoxia**	Hypoxia from mass transfer limitations drives wall degeneration	Marker for regions prone to further pathology
**Flow Topology/Residence Time**	Blood stasis promotes thrombus formation and wall hypoxia	Guides stent design and post-treatment surveillance

**Table 5:** Comprehensive comparison of CFD-based AAA models, their clinical practicality, and limitations. FSI = Fluid–Structure Interaction; ILT = Intraluminal Thrombus; ML = Machine Learning; SVM = Support Vector Machine; KNN = K-Nearest Neighbors; GLM = Generalized Linear Model; GNN = Graph Neural Network; ROM = Reduced Order Model; MD = Maximum Deformation; AI = Artificial Intelligence.

Model/Approach	Key Facts and Features	Practicality for Clinical Use	Main Limitations
**Full Order CFD Models**	High-fidelity hemodynamic/biomechanical simulation; accurate WSS, OSI, PWS, flow patterns; patient-specific geometry	Gold standard for research; not practical for routine clinical use due to high computation	Time-consuming, requires expertise, not real-time, sensitive to modelling assumptions
**Reduced Order Models (ROMs)**	Use machine learning (e.g., GNNs) to approximate CFD results; much faster; maintain reasonable accuracy	Promising for clinical workflows; can enable near real-time predictions	Require high-quality training data; may lose detail/accuracy in complex cases
**ML/AI-Enhanced Models**	Integrate CFD metrics, imaging, and patient data; use SVM, KNN, GLM, deep learning for risk prediction	Emerging; can automate segmentation, risk scoring, and monitoring; some pilot clinical use	Data quality, model explainability, standardization, and regulatory/ethical concerns
**FSI (Fluid–Structure Interaction) Models**	Combine CFD with vessel wall mechanics; account for wall deformation, ILT, material properties	More realistic rupture risk assessment; used in advanced research and select clinical pilots	Complex setup, high computational cost, limited by uncertainty in wall/ILT properties
**Morphological and Multivariate Models**	Combine anatomical (diameter, tortuosity, ILT) and biomechanical indices (PWS, MD) for personalized risk	Nomograms and risk scores can be integrated into clinical decision tools	Need for multicentre validation, may not capture all biological factors
**Ultrasound-Based CFD/FSI**	Uses real-time, non-invasive imaging; enables repeated monitoring; can assess wall mechanics and ILT	Feasible for routine follow-up; less costly and more accessible than CT/MRI	Lower spatial resolution, requires further validation, limited by US image quality
**Blood Flow Pattern Analysis**	Classifies flow (e.g., helical, vortical) via CFD; type III flow linked to high rupture risk	Can improve risk stratification, especially for AAAs < 55 mm; enhances traditional criteria	Requires CFD expertise, not yet standard in clinical protocols
**Bayesian/Patient-Specific Growth Models**	Predicts individual AAA growth using patient data and imaging; improves over population models	Useful for personalized follow-up scheduling and intervention planning	Dependent on longitudinal data, may not directly predict rupture
**Automated CFD Pipelines**	Integrate deep learning for segmentation, open-source tools for workflow; aim to streamline clinical use	Increases accessibility for clinicians, reduces manual workload	Still in development, needs robust validation and user training

**Table 6:** Windkessel effect’s role in CFD biomarker quantification for AAA.

Aspect	Role of Windkessel Effect in CFD	Impact on Biomarker Quantification
**Pathogenesis**	Models compliance/resistance, enabling realistic pressure/flow waveforms	Accurate WSS/OSI mapping for identifying regions of endothelial dysfunction and inflammation
**Progression**	Simulates pressure/flow wave propagation as compliance changes with AAA growth	Refined prediction of PWS, RRT, and thrombus-prone regions
**Post-Treatment**	Accounts for altered compliance after stent grafting	Predicts haemodynamic changes, endoleak risk, and guides device optimization
**Patient-Specific** **Modelling**	Calibrates Windkessel parameters using imaging/clinical data	Enhances accuracy of CFD-derived biomarkers for individualized risk assessment

**Table 7:** Summary of imaging inflammatory biomarkers and how they are obtained from cardiac imaging. CTA = CT Angiography; PCAT/FAI = Pericoronary Adipose Tissue/Fat Attenuation Index; SSTR-PET/CT = Somatostatin Receptor Positron Emission Tomography/Computed Tomography; 18F-FDG PET = 18F-Fluorodeoxyglucose Positron Emission Tomography; DCE-MRI = Dynamic Contrast-Enhanced Magnetic Resonance Imaging.

Biomarker/Metric	Imaging Modality	How It is Obtained/Measured	Clinical Relevance
**VPCI (Perivascular Fat)**	CT Angiography (CTA)	Quantitative analysis of perivascular fat attenuation over time	Predicts reintervention risk post-EVAR, tracks wall inflammation
**PCAT/FAI**	Coronary CTA	Attenuation of pericoronary adipose tissue (HU)	Detects coronary inflammation, predicts cardiac events
**SSTR-PET/CT**	PET/CT	Uptake of somatostatin receptor tracers in inflamed tissue	Maps macrophage-driven inflammation in myocardium
**18F-FDG PET**	PET/CT	Glucose uptake in inflamed vessel/myocardial tissue	Quantifies metabolic activity of inflammation
**DCE-MRI**	Cardiac MRI	Kinetic modelling of contrast agent uptake in vessel wall	Assesses extent of wall inflammation

**Table 8:** Summary of cardiac haemodynamic biomarkers and their predictive value in AAA preoperative assessment.

Biomarker/Parameter	Preoperative Role	Predictive Value for Outcomes
**Low WSS (<0.4 Pa)**	Identifies high-risk patients for rapid expansion/rupture	Independently predicts aneurysm-related events and faster growth
**TAWSS, OSI, RRT**	Detailed mapping of disturbed flow and wall stress	High OSI/RRT linked to inflammation, wall degeneration, rupture
**Combined WSS + Lumen Volume**	Enhanced risk stratification, especially in small AAAs	Outperforms diameter alone for predicting enlargement
**Patient-specific CFD/FSI**	Integrates anatomy and haemodynamics	Personalized risk assessment, guides timing/type of intervention

## Data Availability

No new data were created or analyzed in this study. Data sharing is not applicable to this article.
